# Real-World Experience of Metronomic Chemotherapy in Metastatic Breast Cancer: Results of a Retrospective Unicenter Study

**DOI:** 10.1159/000528042

**Published:** 2023-01-09

**Authors:** Slavomir Krajnak, Jana Krajnakova, Katharina Anic, Katrin Almstedt, Anne-Sophie Heimes, Valerie Catherine Linz, Amelie Loewe, Mona Wanda Schmidt, Annette Hasenburg, Marcus Schmidt, Marco Johannes Battista

**Affiliations:** Department of Gynecology and Obstetrics, University Medical Center of the Johannes Gutenberg-University Mainz, Mainz, Germany

**Keywords:** Metronomic chemotherapy, Metastatic breast cancer, Disease control rate, Survival, Toxicity

## Abstract

**Introduction:**

Metronomic chemotherapy (MCT) is increasingly used in oncology due to its favorable therapeutic index. There is still a lack of evidence for MCT in metastatic breast cancer (MBC). In this retrospective unicenter study, we demonstrated real-word data on MCT in MBC.

**Methods:**

MBC patients who received metronomic oral cyclophosphamide (CTX) (50 mg daily) and methotrexate (MTX) (2.5 mg every other day), CTX and capecitabine (CAPE) (500 mg thrice daily), CTX, or vinorelbine (VRL) (30 mg daily) alone for at least 4 weeks between 2009 and 2021 were included. The primary endpoint was disease control rate (DCR) ≥24 weeks. Secondary endpoints were progression-free survival (PFS) and overall survival (OS). Patient characteristics and therapy response were analyzed using χ^2^ test. For survival analyses, Kaplan-Meier estimator and log-rank test were used.

**Results:**

Seventy-two patients were identified. Sixty-two patients received CTX/MTX, three CTX/CAPE, two CTX, and five VRL. Median age at diagnosis MBC and at start of MCT was 59.0 years and 64.5 years, respectively. 72.2% tumors were hormone receptor positive and 27.8% were triple-negative. 54.2% patients had more than two different metastases. 80.6% patients showed visceral involvement. 31.9% patients achieved DCR ≥24 weeks. Median PFS was 17.0 weeks (95% CI 14.5–19.5) and median OS was 58.0 weeks (95% CI 29.0–87.0). MCT showed similar DCR ≥24 weeks and clinically meaningful but not statistically significant shorter median PFS compared to prior therapy (31.9% versus 32.8% [*p* = 0.570] and 17.0 weeks versus 20.0 weeks [*p* = 0.093], respectively) and statistically significant higher DCR ≥24 weeks and longer median PFS compared to subsequent therapy (31.9% versus 17.4% [*p* = 0.038] and 17.0 weeks versus 12.0 weeks [*p* = 0.006], respectively). Three (4.2%) patients terminated MCT because of toxicity.

**Conclusion:**

In this real-world retrospective study, MCT was effective and well tolerated and may thus represent a valuable treatment option in selected MBC patients.

## Introduction

Metastatic breast cancer (MBC) represents one of the leading cause of cancer mortality worldwide [1]. In general, MBC is an incurable disease with a median overall survival (OS) of about 3 years and a 5-year survival rate of about 25% [2, 3]. Hormone receptor (HR), human epidermal growth factor receptor 2 (HER2), breast cancer 1/2 gene (BRCA 1/2), phosphatidylinositol-4,5-bisphosphate 3-kinase catalytic subunit alpha, programmed death-ligand 1 status, as well as biological age, tumor burden, and prior therapies critically influence prognosis [2, 3, 4]. Considering these prognostic factors, the therapeutic goal is to achieve chronification of the disease with a prolongation of survival and preservation of quality of life [5].

Metronomic chemotherapy (MCT), defined as a chronic administration of conventional chemotherapeutic agents at low doses without prolonged drug-free breaks, was first mentioned by Hanahan and Kerbel in early 2000 [6, 7]. Since then, numerous studies with MCT have shown promising results. Most of the data originate from phase 2 and retrospective trials, with breast cancer being the most studied tumor entity. As there is growing evidence of efficacy and good tolerability, MCT is increasingly appreciated as a possible treatment option for MBC [2, 3]. Oral administration of MCT is safe and popular among patients because of its ease of use and flexibility of drug dosing in case of toxicities [8, 9, 10]. Dose accumulations associated with intolerable side effects are rare, so the medication can be administered for longer periods of time [11]. The mechanisms of action are not truly cytotoxic but rather multimodal, particularly via inhibition of angiogenesis, immunomodulation, and effects on tumor stroma [6, 7, 12, 13]. It is assumed that MCT is not simply a different way of administering chemotherapy (CT) but a truly new treatment option [14, 15]. Nevertheless, the data are still insufficient to identify which patients will benefit most and which agent or combination is most appropriate. This retrospective study aimed to investigate the efficacy and toxicity of MCT in real-world settings, taking into account the treatment prior and subsequent to MCT as well as clinicopathological parameters.

## Materials and Methods

### Study Population and Treatment

MBC patients who received MCT in the form of oral cyclophosphamide (CTX) (50 mg daily) and methotrexate (MTX) (2.5 mg every other day), CTX (50 mg daily) and capecitabine (CAPE) (500 mg thrice daily), CTX (50 mg daily) alone, or vinorelbine (VRL) (30 mg daily) for at least 4 weeks between February 2009 and December 2021 at the University Medical Center Mainz were selected for this retrospective analysis. Patients with HER2-positive tumors and patients with presence of additional cancer were excluded. No antiemetic treatment was routinely given to patients during MCT. Clinicopathological and follow-up data until March 2022 were collected as previously reported by our group [16]. Patients who had not progressed or died by the cutoff date of March 2022 were censored. The manuscript was written in accordance with the STROBE Statement checklist for cohort studies of the EQUATOR network reporting guidelines [17].

### Clinical Outcomes

The primary endpoint was disease control rate (DCR) ≥24 weeks. DCR included stable disease, partial response, and complete response. Secondary endpoints were progression-free survival (PFS) and OS. Disease-free interval was defined as the time from completion of primary therapy for early breast cancer to the evidence of recurrence or metastatic disease. The therapy efficacy was assessed using the standard clinical and imaging methods. Assessment of safety and tolerability of MCT was conducted according to the National Cancer Institute Common Terminology Criteria for Adverse Events (NCI CTCAE) version 4.03. For subgroup analyses, we stratified the patients by age at start of MCT (younger: ≤ median age vs. older: > median age), HR status (HR positive: estrogen/progesterone receptor positive and HER2 negative vs. triple negative: estrogen/progesterone receptor negative and HER2 negative), number of different metastatic sites (without multiple metastases: ≤2 different metastatic sites vs. multiple metastases: >2 different metastatic sites), and number of prior CT lines (nonheavily pretreated: <2 CT lines vs. heavily pretreated: ≥2 CT lines).

### Statistical Analyses

Statistical analyses were performed using the SPSS statistical software system, version 27.0 (SPSS Inc., Chicago, IL, USA). Patient characteristics were analyzed descriptively using median and range for continuous data and relative and absolute frequencies for categorical data. The associations between clinicopathological characteristics and therapy response were analyzed by applying a Pearson's χ^2^ test. For PFS and OS analyses, Kaplan-Meier estimator and log-rank test were used. All tests were two sided and a *p* < 0.05 was considered statistically significant.

## Results

### Patient Characteristics

A total of 72 patients were included into the study (Table 1). Conventional CT was administered in 40 (59.7%) patients as the main therapy immediately prior to MCT and in 36 (78.3%) patients subsequent to MCT. Five (6.9%) patients received MCT as first-line therapy and 62 (86.1%) patients showed progressive disease from prior treatment at the start of MCT. No concomitant treatment such as radiotherapy, endocrine or targeted therapy was observed in this cohort.

### Therapy Response

DCR ≥24 weeks was achieved in 23 (31.9%) patients (Table 2). Median PFS and OS were 17.0 weeks (95% confidence interval [CI] 14.5–19.5) and 58.0 weeks (95% CI 29.0–87.0), respectively. CTX/MTX was the most common metronomic regime with DCR ≥24 weeks of 30.6%. There were no significant differences in DCR and survival between different metronomic regimens, with 62, 3, 2, and 5 patients receiving CTX/MTX, CTX/CAPE, CTX, and VRL, respectively. Compared to MCT, therapy prior to MCT showed a similar DCR ≥24 weeks of 32.8% (*p* = 0.570) and therapy subsequent to MCT showed a significantly lower DCR ≥24 weeks of 17.4% (*p* = 0.038) (Table 2).

**Fig. 1:**
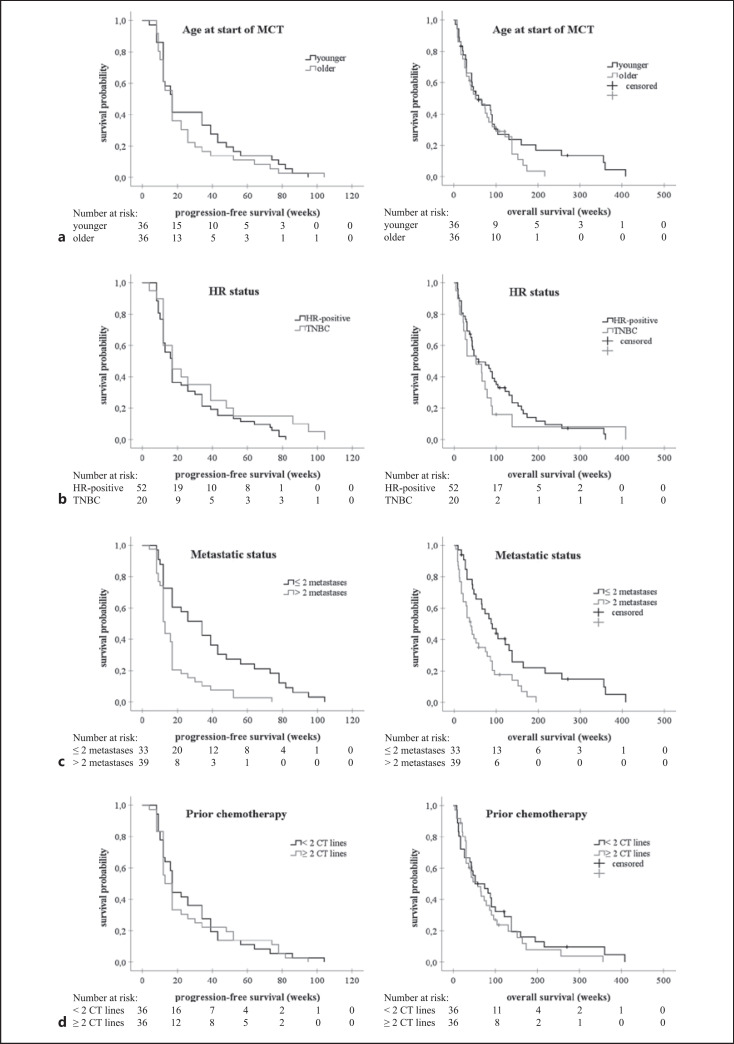
Kaplan-Meier analyses of PFS and OS regarding age at start of MCT (**a**), HR status (**b**), number of different metastatic sites (**c**), and number of prior CT lines (**d**). **a** Younger versus older: median PFS: both 17.0 weeks, log rank: *p* = 0.389, median OS: 58.0 weeks versus 52.0 weeks, log rank: *p* = 0.237. **b** HR positive versus TNBC: median PFS: both 17.0 weeks, log rank: *p* = 0.144, median OS: 58.0 weeks versus 52.0 weeks, log rank: *p* = 0.379. **c** ≤2 metastases versus >2 metastases: median PFS: 34.0 weeks versus 13.0 weeks, log rank: *p* < 0.001, median OS: 91.0 weeks versus 39.0 weeks, log rank: *p* = 0.003. **d** <2 prior CT lines versus ≥2 prior CT lines: median PFS: 17.0 weeks versus 13.0 weeks, log rank: *p* = 0.764, median OS: 52.0 weeks versus 58.0 weeks, log rank: *p* = 0.457. MCT, metronomic chemotherapy; HR, hormone receptor; TNBC, triple-negative breast cancer; PFS, progression-free survival; OS, overall survival; CT, chemotherapy.

Regarding age, median PFS (17.0 weeks in both younger and older patients) and OS (58.0 weeks in younger vs. 52.0 weeks in older patients) did not show any significant differences (*p* = 0.389 and *p* = 0.237, respectively) (Fig. 1a). Median PFS in both HR-positive and triple-negative subgroup was 17.0 weeks (*p* = 0.144) (Fig. 1b). Median OS was 58.0 weeks (95% CI 17.2–98.8) in patients with HR-positive disease and 52.0 weeks (95% CI 11.0–93.0) in patients with triple-negative breast cancer (TNBC) (*p* = 0.379). Patients without multiple metastases had significantly longer median PFS and OS than those with multiple metastases (34.0 weeks [95% CI 20.6–47.4] vs. 13.0 weeks [95% CI 11.5–14.5], *p* < 0.001, and 91.0 weeks [95% CI 53.7–128.3] vs. 39.0 weeks [95% CI 26.1–51.9], *p* = 0.003, respectively) (Fig. 1c). Median PFS and OS did not differ between nonheavily pretreated and heavily pretreated patients (17.0 weeks [95% CI 15.8–18.2] vs. 13.0 weeks [95% CI 9.3–16.7], *p* = 0.764, and 52.0 weeks [95% CI 2.0–102.0] vs. 58.0 weeks [95% CI 31.4–84.6], *p* = 0.457, respectively) (Fig. 1d). In terms of prior and subsequent therapy, MCT showed a clinically relevant but not statistically significant shorter median PFS compared to prior therapy (17.0 weeks vs. 20.0 weeks, *p* = 0.093) and a significantly longer median PFS compared to subsequent therapy (17.0 weeks vs. 12.0 weeks, *p* = 0.006) (Table 2).

### Safety Results

The most common adverse events leading to discontinuation of MCT (≥ grade 3) were nausea/vomiting and diarrhea (2 patients) and thrombocytopenia (1 patient) (Table 3). In the CTX/MTX group, 5 (8.1%) patients discontinued MTX without termination of CTX. Two (3.2%) patients terminated MTX due to nausea/vomiting and each (1.6%) patient due to fatigue, mucositis, and vision impairment, respectively. Neutropenia or anemia ≥ grade 3 did not occur in our cohort. Overall, MCT was terminated early due to toxicity in 3 (4.2%) patients and low performance status in 7 (9.7%) patients, which was similar compared with prior and subsequent treatment (7.5% and 10.9% due to toxicity and 0.0% and 10.9% due to low performance status in prior and subsequent therapy, respectively).

## Discussion

In this retrospective unicenter study analyzing MCT in real-world settings, CTX/MTX was the most common metronomic regimen, accounting for 86.1% of all patients, followed by VRL in 6.9%, CTX/CAPE in 4.2%, and CTX in 2.8% patients. No concomitant treatment such as radiotherapy, endocrine or targeted therapy was given in our cohort. In the largest multicenter retrospective study VICTOR-6, which collected data of 597 MBC patients who received MCT between January 2011 and December 2016 in 43 Italian Oncology sites, most patients (79.3%) received MCT as single agent (VRL 34.6%, CAPE 22.3%, and CTX 20.7%). The use of VRL- and CTX-based regimens increased during the observation period (2011: 16.8% and 17.1%, 2016: 29.8% and 25.6%, respectively) [11]. In a meta-analysis conducted by Liu et al. [18], subgroup analysis did not show any significant difference in the clinical benefit rate (CBR) among different metronomic agents as well as between MCT alone and the combination regimens. In the present study, there were also no significant differences regarding DCR and survival among the different metronomic regimens.

The primary endpoint DCR ≥24 weeks was 31.9%, which was in line with the results of previous studies [11, 19, 20, 21]. In the VICTOR-6 study, the relatively high DCR of 74.4% could be attributed to the fact that patients were included into the study regardless of their response to prior treatment. In addition, the definition of the timing of DCR was not clearly specified. Finally, the patient populations studied were very heterogeneous and can only be compared with caution [11]. In our analysis, which included only patients with disease progression or intolerable toxicity from prior treatment, CTX/MTX was the most common metronomic regimen with a DCR ≥24 weeks of 30.6%. The results were consistent with a DCR ≥24 weeks of 31.2% in the Chinese population, 40.3% of whom received CTX/MTX as maintenance therapy without disease progression from previous treatment [20]. Survival analyses revealed median PFS and OS of 17.0 weeks and 58.0 weeks, respectively, which was consistent with the results of previous studies [11, 20, 22, 23].

In the subgroup analyses, we observed a significantly longer median PFS (34.0 weeks vs. 13.0 weeks, *p* < 0.001) and OS (91.0 weeks vs. 39.0 weeks, *p* = 0.003) in the subgroup without multiple metastases compared to the subgroup with multiple metastases. It can be assumed that patients with few metastases are more likely to have less aggressive disease with a lower probability of extensive visceral involvement. We did not find any significant differences regarding age at start of MCT, HR status, and CT line in survival analyses. In the VICTOR-1 study, metronomic combination of VRL and CAPE had an acceptable efficacy profile (overall response rate 33%, CBR 67%) and was well tolerated in MBC patients aged ≥70 years [24]. Also in older and frail HER2-positive MBC patients, addition of metronomic CTX to trastuzumab plus pertuzumab increased median PFS by 7 months compared to dual HER2 blockade alone and was relatively well tolerated [25]. Patients with HR-positive, HER2-negative metastatic disease resistant to endocrine-based therapy and who do not require rapid tumor response are generally suitable for MCT [2, 14]. However, MCT may also be promising in patients with TNBC, as demonstrated in the current analysis, which did not show any significant differences in survival between the HR-positive and the TNBC cohort. In the subgroup analysis of the VICTOR-6 study, patients with triple-negative MBC showed a median PFS, OS, and DCR of 6.0 months (95% CI 4.9–7.2), 12.1 months (95% CI 9.6–16.7), and 64.9%, respectively [26]. In the present study, similar to most trials, patients with HER2-positive tumors were excluded. In recent years, there has been an increasing use of the combination of MCT with anti-HER2 therapies. In the HEX trial, the combination of standard trastuzumab and metronomic CTX (50 mg once daily) plus CAPE (500 mg thrice daily) in 60 patients with untreated HER2-positive MBC demonstrated favorable efficacy with a median PFS of 11.0 months (95% CI 6.3–15.6) and a CBR of 78.2% [27]. Also, the combination of metronomic VRL (40 mg thrice weekly) and trastuzumab showed activity with a median PFS of 7.4 months (95% CI 3.2–11.5) and a CBR of 75% [28].

MCT showed similar DCR ≥24 weeks and clinically meaningful but not statistically significant shorter median PFS compared to prior therapy (31.9% vs. 32.8% [*p* = 0.570] and 17.0 weeks vs. 20.0 weeks [*p* = 0.093], respectively) and statistically significant higher DCR ≥24 weeks and longer median PFS compared to subsequent therapy (31.9% vs. 17.4% [*p* = 0.038] and 17.0 weeks vs. 12.0 weeks [*p* = 0.006], respectively). Based on these results, it can be assumed that MCT was as effective as standard prior therapy and that patients experienced significant disease progression during subsequent therapy. It is important to note that MCT was administered in 5 (6.9%) patients in first-line setting and 24 (33.3%) patients received no further therapy after MCT because of low performance status or death. Nearly 20% of patients terminated MCT due to low performance status or death. This suggests that there were many frail and pretreated patients in our cohort, so this should be taken into consideration when interpreting and comparing the results with other studies. The extent to which the efficacy of MCT, as illustrated by comparable DCR ≥24 weeks and median PFS with the prior treatment line, might improve OS remains speculative because of several limitations. This single-arm study was designed to describe the experience of a single institution. A cross-study comparison with historical cohorts of MBC is difficult due to the heterogeneity of the cohort, which included patients in first-line setting as well as heavily pretreated patients who were treated with different agents.

MCT was well tolerated. Only 3 (4.2%) patients terminated MCT due to toxicity, which was similar compared with prior and subsequent treatment. The favorable toxicity profile of MCT with similar results has also been demonstrated in previous studies in which 2.2–14.9% of patients discontinued MCT due to toxicity [11, 20, 23, 29]. Compared with conventional CT, MCT is associated with fewer adverse events such as polyneuropathy, myelosuppression, mucositis, and hair loss and may thus contribute substantially to the maintenance of quality of life, which is one of the main goals of treatment at this stage [19, 30]. However, to date, data allowing a direct comparison between MCT and conventional CT in terms of tolerability and quality of life are lacking.

There were limitations in the present study. The retrospective design can lead to selection bias, so the interpretation of the presented results is limited. The vast majority of patients received MCT before or at the time of introduction of cyclin-dependent kinase 4/6 inhibitors for HR-positive, HER2-negative MBC and pembrolizumab for triple-negative MBC. Available studies on MCT often show small, heterogeneous patient populations with different agents and different doses. In addition, the metronomic agents are often combined with each other as well as with other substances. It is therefore difficult to interpret the presented data and the exact role of MCT in terms of current recommendations for MBC treatment. Parts of the clinical data from our MCT cohort were already published elsewhere [16, 31, 32]. In this study, MCT patients were included regardless of the active agent, and OS data were presented for the first time.

## Conclusion

This real-world retrospective unicenter study on MCT showed favorable efficacy with minimal toxicity in MBC patients. The data presented support the premise that MCT is appreciated as a valuable treatment option in selected patients. However, to establish the appropriate role of MCT in the treatment algorithm for MBC and to identify the most appropriate patient population, no effort should be spared to test the efficacy and safety of MCT in randomized controlled trials compared with modern treatment regimens.

## Statement of Ethics

All procedures performed in this study were in accordance with the ethical standards of the institutional and national research committee and with the World Medical Association Declaration of Helsinki. Data collected in this study were obtained in the context of routine medical care. Ethical approval for the use of data for research purposes as well as consent for participation and publication were not required for this study in accordance with the Ethics Committee of the Landesaerztekammer Rheinland-Pfalz, Germany. Written informed consent from participants was not required in accordance with local/national guidelines.

## Conflict of Interest Statement

Slavomir Krajnak: lecture: Novartis and Roche. Research funding: Novartis. Travel reimbursement: Novartis and PharmaMar. Katharina Anic: lecture: AstraZeneca, Clovis Oncology, and MSD. Katrin Almstedt: lecture: AstraZeneca, Pfizer, and Roche. Anne-Sophie Heimes: lecture: Medupdate GmbH, Pfizer, and Roche. Annette Hasenburg: advisory board: AstraZeneca, GSK, LEO Pharma, MSD, PharmaMar, Roche, and Tesaro. Lecture: AstraZeneca, Celgene, Clovis Oncology, LEO Pharma, MedConcept GmbH, Med update GmbH, Medicultus, Pfizer, PharmaMar, Roche, Streamedup! GmbH, and Tesaro. Marcus Schmidt: lecture: AstraZeneca, BioNTech, Daiichi Sankyo, Eisai, Lilly, MSD, Novartis, Pantarhei Bioscience, Pfizer, Pierre Fabre, Roche, and SeaGen. Research funding: AstraZeneca, BioNTech, Eisai, Genentech, Novartis, Pantarhei Bioscience, Pfizer, Pierre-Fabre, Roche, and SeaGen. Travel reimbursement: Pfizer and Roche. Marco Johannes Battista: advisory board: Eisai, GSK, MSD, PharmaMar, Roche, and Tesaro. Lectures: Astra Zeneca, Clovis Oncology, GSK, MSD, PharmaMar, Roche, and Tesaro. Research funding: AstraZeneca, Clovis Oncology, MSD, and Novartis. All other authors have no conflicts of interest to declare.

## Funding Sources

No funding was received.

## Author Contributions

Conceptualization and methodology: Slavomir Krajnak, Marcus Schmidt, and Marco Johannes Battista; formal analysis and investigation: Slavomir Krajnak and Jana Krajnakova; writing − original draft: Slavomir Krajnak; writing − review and editing: Slavomir Krajnak, Jana Krajnakova, Katharina Anic, Katrin Almstedt, Anne-Sophie Heimes, Valerie Catherine Linz, Amelie Loewe, Mona Wanda Schmidt, Annette Hasenburg, Marcus Schmidt, and Marco Johannes Battista. All authors read and approved the final manuscript.

## Data Availability Statement

The datasets generated during the current study are available from the corresponding author on reasonable request.

## Figures and Tables

**Table 1:** Patient characteristics

Characteristics	Patients (*n* = 72), *n* (%)
Age at diagnosis of BC, years	
Median	50.5
Range	29.0–80.0
Age at diagnosis of MBC, years	
Median	59.0
Range	33.0–86.0
Age at start of MCT, years	
Median	64.5
Range	35.0–87.0
Time between diagnosis of MBC and start of MCT	
≤2 years	31 (43.1)
≤5 years	24 (33.3)
>5 years	17 (23.6)
HR status	
HR positive	52 (72.2)
HR negative (TNBC)	20 (27.8)
Disease status	
De novo MBC	10 (13.9)
DFI ≤5 years	20 (27.8)
DFI ≤10 years	21 (29.2)
DFI >10 years	21 (29.2)
Metastatic sites	
Median	3.0
Range	1–7
Number of metastatic sites	
≤2	33 (45.8)
>2	39 (54.2)
Metastatic sites	
Bone	49 (68.1)
Liver	35 (48.6)
Lung	34 (47.2)
Pleura	20 (27.8)
Peritoneum	13 (18.1)
Distant lymph nodes	36 (50.0)
Cerebrum	7 (9.7)
Soft Tissue	16 (22.2)
Prior therapy for metastatic disease	
CT	
Median	1.5
Range	0–6
Lines of CT	
<2	36 (50.0)
≥2	36 (50.0)
Endocrine therapy	
Median	2.0
Range	0–7
Lines of endocrine therapy	
<2	28 (38.9)
≥2	44 (61.1)
Agents for endocrine therapy	
SERM	11 (15.3)
SERD	41 (56.9)
AI nonsteroidal	42 (58.3)
AI steroidal	31 (43.1)
GnRH agonist	6 (8.3)
Targeted therapy	
None	40 (55.6)
Anti-HER2 therapy	3 (4.2)
VEGF inhibitor	14 (19.2)
PD-L1 inhibitor	1 (1.4)
mTOR inhibitor	20 (27.8)
CDK 4/6 inhibitor	10 (13.9)
PARP inhibitor	1 (1.4)
Anti-resorptive therapy	
None	25 (34.7)
Bisphosphonates	31 (43.1)
Denosumab	20 (27.8)
Radiotherapy	
None	26 (36.1)
Bone	39 (54.2)
Cerebrum	7 (9.7)

MCT, metronomic chemotherapy; BC, breast cancer; MBC, metastatic breast cancer; HR, hormone receptor; TNBC, triple-negative breast cancer; DFI, disease-free interval; SERM, selective estrogen receptor modulator; SERD, selective estrogen receptor degrader; AI, aromatase inhibitor; GnRH, gonadotropin-releasing hormone; HER2, human epidermal growth factor receptor 2; VEGF, vascular endothelial growth factor; PD-L1, programmed death-ligand 1; mTOR, mammalian target of rapamycin; CDK, cyclin-dependent kinase; PARP, poly-ADP-ribose polymerase.

**Table 2:** Therapy response to MCT and its prior and subsequent therapy

Therapy	MCT overall	CTX/MTX	CTX/CAPE	CTX	VRL	Therapy prior to MCT	Therapy subsequent to MCT
Patients, *n* (%)	72	62 (86.1)	3 (4.2)	2 (2.8)	5 (6.9)	67	46
DCR ≥24 weeks, *n* (%)	23 (31.9)	19 (30.6)	1 (33.3)	1 (50.0)	2 (40.0)	22 (32.8)	8 (17.4)
*p* value	0.919					0.570	0.038
SD/PR/CR (≥24 weeks), *n* (%)	15 (20.8)/7 (9.7)/1 (1.4)	11 (17.7)/7 (11.3)/1 (1.6)	1 (33.31/0 (0.0)/0 (0.0)	1 (50.01/0 (0.0)/0 (0.0)	2 (40.01/0 (0.0)/0 (0.0)	14 (20.9)/8 (11.9)/0 (0.0)	8 (17.4)/0 (0.0)/0 (0.0)
*p* value	0.932					0.763	0.001
PFS (weeks) (median, 95% CI)	17.0 (14.5-19.5)	17.0 (13.9-20.1)	17.0 (9.0-25.0)	4.0 (N/A)	17.0 (15.9-18.1)	20.0 (16.6-23.4)	12.0 (9.1-14.9)
*p* value	0.410					0.093	0.006
OS (weeks) (median, 95% CI)	58.0 (29.0-87.0)	47.0 (25.7-68.3)	138.0 (N/A)	4.0 (N/A)	160.0 (112.8-207.2)	N/A	N/A
*p* value	0.127					N/A	N/A

CTX, cyclophosphamide; MTX, methotrexate; CAPE, capecitabine; VRL, vinorelbine; DCR, disease control rate; SD, stable disease; PR, partial response; CR, complete response; CI, confidence interval; PFS, progression-free survival; OS, overall survival.

**a:** Adverse events leading to discontinuation of MCT

MCT agent		Patients (*n* = 72), *n* (%)				
CTX/MTX		62				
	PD	46 (74.2)				
	Death	6 (9.7)				
	Low performance status	7 (11.3)				
	Nausea/vomiting and diarrhea	2 (3.2)				
	Thrombocytopenia	1 (1.6)				
MTX alone (CTX not terminated)	Fatigue	1 (1.6)				
	Nausea/vomiting	2 (3.2)				
	Mucositis	1 (1.6)				
	Vision impairment	1 (1.6)				
CTX/CAPE		3				
	PD	3 (100.0)				
CTX		2				
	PD	1 (50.0)				
	Death	1 (50.0)				
VRL		5				
	PD	5 (100.0)				

**b:** Termination of MCT compared to prior and subsequent therapy

Therapy		Therapy prior to MCT	MCT	*p* value	Therapy subsequent to MCT	*p* value
Patients, *n*		67	72		46	
Therapy termination	PD	62 (92.5)	55 (76.4)	0.255	28 (60.9)	0.486
	Death	0 (0.0)	7 (9.7)		7 (15.2)	
	Toxicity	5 (7.5)	3 (4.2)		5 (10.9)	
	Low performance status	0 (0.0)	7 (9.7)		5 (10.9)	
	Without termination	0 (0.0)	0 (0.0)		1 (2.2)	

MCT, metronomic chemotherapy; CTX, cyclophosphamide; MTX, methotrexate; PD, progressive disease; CAPE, capecitabine; VRL, vinorelbine.
